# Supplementary material for: Chromothripsis during telomere crisis is independent of NHEJ, and consistent with a replicative origin
Source: Genome Res. 2019 May;29(5):737–49. doi: 10.1101/gr.240705.118 (PMC6499312; doi:10.1101/gr.240705.118)
Supplement: Supplemental Material [file supp_gr.240705.118_Supplemental_file_1.zip › contigs/annotated_contigs/DB53/contig.2.DB53_length_273_mean_cov_1.96703296703.docx]

**DB53_length_273_mean_cov_1.96703296703**

```
GAATTCAAGGTCATGAAGACATTATTTAACCTTTTAGAATATTTTATATAGCTTAGAGTACACAATGAAGTAGGGGAAATTAGTGATTA
 >chr8:79150933-79151098 - E=4e-85 p=0e+00
AGAAATCATTTATTATTATATATTT|TTATTATACTTTAAGTTCTAGGGTACATGTGCACAACGTGCAGGTTTGTTA|CATCGGTATAC
                          >chr17:68796206-68796338 - E=4e-44
ATGTGCTGTGCTGGTGTGCTGCACCCATCAACTCTTCATTTACATTCAGTCTTGCTCCTAATGGTATCCC|CCACCCTACCCGTCACGC

CACTACGGA
```
